# High-fidelity eye, head, body, and world tracking with a wearable device

**DOI:** 10.3758/s13428-022-01888-3

**Published:** 2022-07-25

**Authors:** Vasha DuTell, Agostino Gibaldi, Giulia Focarelli, Bruno A. Olshausen, Martin S. Banks

**Affiliations:** 1 Wertheim School of Optometry and Vision Science, UC Berkeley, Minor Hall, Berkeley, CA USA; 2 Redwood Center for Theoretical Neuroscience, UC Berkeley, Evans Hall, Berkeley, CA USA

**Keywords:** Eye tracking, Human vision, Hardware, Natural scene statistics

## Abstract

We describe the design and performance of a high-fidelity wearable head-, body-, and eye-tracking system that offers significant improvement over previous such devices. This device’s sensors include a binocular eye tracker, an RGB-D scene camera, a high-frame-rate scene camera, and two visual odometry sensors, for a total of ten cameras, which we synchronize and record from with a data rate of over 700 MB/s. The sensors are operated by a mini-PC optimized for fast data collection, and powered by a small battery pack. The device records a subject’s eye, head, and body positions, simultaneously with RGB and depth data from the subject’s visual environment, measured with high spatial and temporal resolution. The headset weighs only 1.4 kg, and the backpack with batteries 3.9 kg. The device can be comfortably worn by the subject, allowing a high degree of mobility. Together, this system overcomes many limitations of previous such systems, allowing high-fidelity characterization of the dynamics of natural vision.

## Introduction

The visual system evolved and developed in the natural environment, so obtaining a full understanding of its function requires studying how vision is engaged in everyday tasks. For this reason, there is a great need to expand vision science beyond the controlled laboratory setting and into the natural world. Data collected in such natural conditions provide crucial information about mechanisms underlying stereopsis (Liu, Bovik, & Cormack, 2008; Sprague, Cooper, Tošić, & Banks, 2015; Gibaldi & Banks, 2019; Gibaldi & Banks, 2021), eye movements (Gibaldi & Banks, 2019) and their coordination with head movements (Kothari et al., 2020; Hausamann, Sinnott, & MacNeilage, 2020; Land & Hayhoe, 2001), eye optics (Gibaldi, Labhishetty, Thibos, & Banks, 2021), and other motor behaviors (Matthis et al., 2018; Bonnen et al., 2019, 2021). To create a better account of natural sensory-motor relationships, data must be collected along with eye tracking, depth, and motion information when the subject performs everyday tasks in the real world. Furthermore, many applications, such as measurement of the power spectrum (DuTell, Gibaldi, Focarelli, Olshausen, & Banks, 2020), require data to be recorded with high spatial and temporal resolution but without compression artifacts. Designing and building a device that fits these requirements presents many serious technical challenges. We first review previous work and then describe our device.

Early work in mobile eye tracking was restricted to the indoor laboratory environment: for instance using hard-wired acquisition computers and coil-based eye tracking (Grossman, Leigh, Abel, Lanska, & Thurston, 1988). Later work pioneered the collection of real-world scene and gaze-tracking data, adapting eye-tracking hardware designed for use in the laboratory into devices that allowed mobile recording outside the lab (Imai, Moore, Raphan, & Cohen, 2001; Einhäuser et al., 2007; Liu et al., 2008; Yamada et al., 2010; Sprague et al., 2015; Gibaldi & Banks, 2019). Unfortunately, cameras in these devices had very limited spatial and temporal resolution, and heavy and bulky eye-tracking hardware limited subject mobility.

More recent efforts utilized compact hardware that is amenable to mobile data collection outside the lab; see Cognolato, Atzori, and Müller (2018) for a recent review. In particular, the introduction of lightweight, mobile-friendly eye trackers such as the Pupil Labs system (Kassner, Patera, & Bulling, 2014) and Tobii glasses (Tobii Pro AB, 2014), as well as lightweight sensors such as Intel RealSense devices (Keselman, Iselin Woodfill, Grunnet-Jepsen, & Bhowmik, 2017), has led to more work in this area (Matthis, Yates, & Hayhoe, 2018; Shankar, Sinnott, Binaee, Lescroart, & MacNeilage, 2021; Solbach & Tsotsos, 2021). In addition, improved usability of collection software has allowed collection of hundreds of hours of data for many subjects (Valsecchi, Akbarinia, Gil-Rodriguez, & Gegenfurtner, 2020; Shankar et al., 2021). However, these datasets offer only low-to-medium temporal resolution and medium-to-high spatial resolution because of the limited capabilities of the scene cameras. An exception is the high-resolution data reported by Emery, Zannoli, Warren, Xiao, and Talathi (2021); but this is for subjects navigating virtual environments. Many of the previous devices also employ cameras with on-device H.264/H.265 encoding, which introduces compression artifacts into the data.

We present a solution to these issues with a wearable device optimized to obtain robust, high-fidelity, multi-modal data, while remaining lightweight and portable enough to enable data collection during everyday behavior in the natural environment. Our solution adapts consumer electronics and laboratory hardware to the needs of mobile, head-mounted tracking. The hardware is combined with custom software that enables accurate, high-resolution data acquisition and post-processing with a convenient interface.

## Hardware

### Devices and sensors

**Fig. 1:**
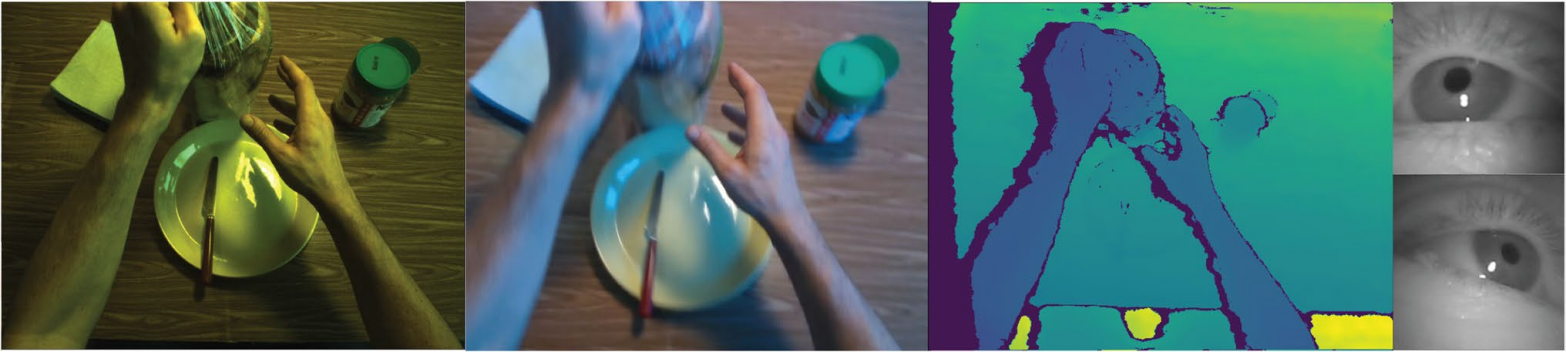
*Left to right*: Sample frames collected from XIMEA camera, RealSense D435i RGB stream, RealSense D435i depth stream, and Pupil Labs binocular eye-tracking cameras. Images shown are frames as captured by each sensor, before post-processing

To record information from the subject and scene, our device uses six sensors (Table 1). To capture high-fidelity video, we use a XIMEA PCIE RGB camera with a global shutter running at 200 Hz. The configuration shown uses a lens offering a 61^∘^× 46^∘^ field of view, but this is easily changeable with a different lens. We supplement this color video with corresponding depth information by including an Intel RealSense D435i, which records both depth and RGB video streams (Fig. 1).

**Table 1:** Device sensors and settings utilized by the system

Device	Resolution	FoV	Model	Location	Data format	Accuracy
High-Fidelity RGB Camera	2064 × 1544 @ 200Hz Global Shutter	Variable (61 x 46)	XIMEA MX031CGSYX2G2-FL	Head	8-bit CMYK Raw binary	–
RGB-D Camera	RGB: 640 × 480 @ 60Hz D: 848 x 480 @ 90Hz	64^∘^× 41^∘^ 86^∘^× 57	RealSense D435I	Head	MPEG-4 NumPy/PNG	D: 2*%* at 2m
				–	NumPy/PNG	–
Binocular Eye Tracker	192 × 192 @ 200Hz	37^∘^× 37^∘^	Pupil Labs	L/R Eye	MPEG-4	0.60^∘^ (Pupil Labs Algorithm)^∘^
Odometry Tracker 1	200 Hz	163 ± 5^∘^	RealSense T265	Head	.pldata	< 1*%* drift
Odometry Tracker 2	200 Hz	–	RealSense T265	Body	.pldata	< 1*%* drift

These settings yield the best overall results for our experimental setup, but resolution and frame-rate settings for the RGB-D and eye-tracking cameras can be easily modified in the GUI. The XIMEA camera’s spatial and temporal resolution is easily changed in a YAML file, and the field of view modified with a lens change

**Fig. 2:**
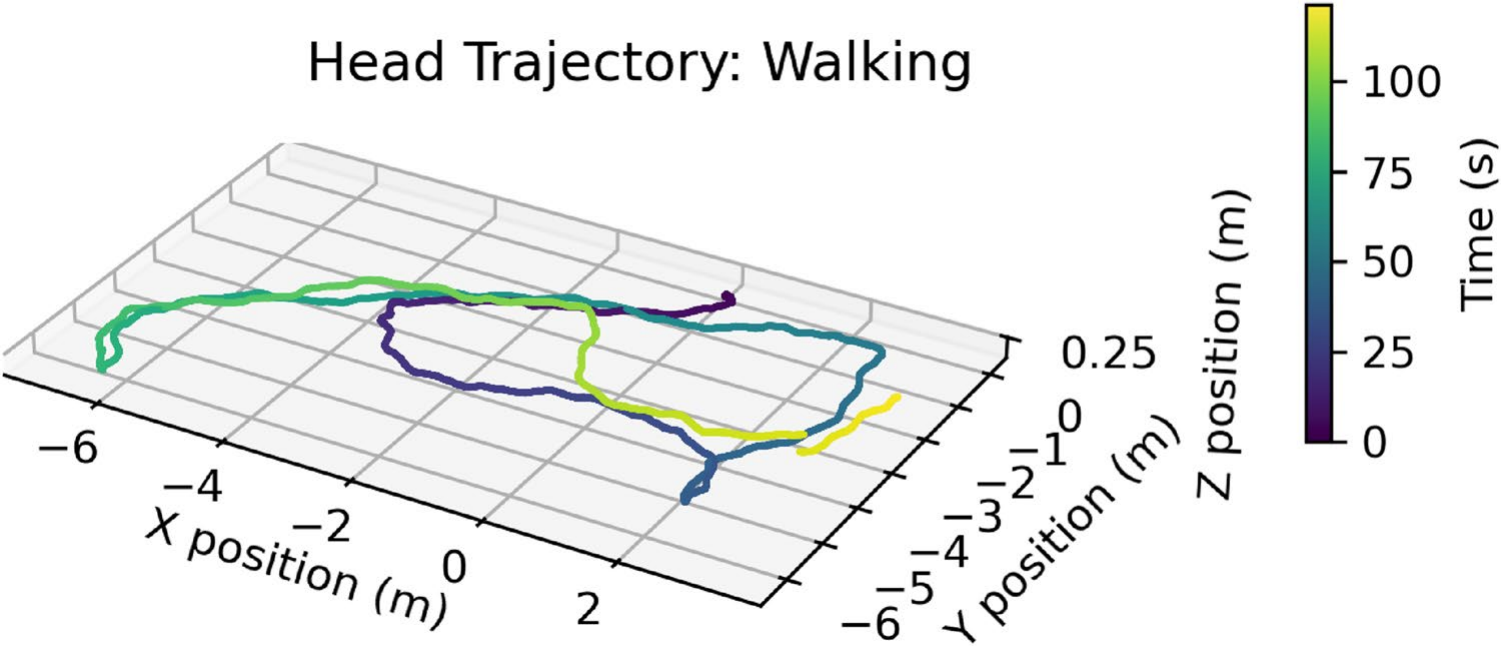
Example trajectory of head position as a person walks through an indoor environment. Color evolves over time from purple to yellow over 2 min. A RealSense T265 tracking sensor collects head position data (such as this one) along with orientation and velocity data at 200 Hz. Another tracker placed on the body provides odometry information for the body

Our device allows us to match the high-fidelity world-camera data to a lower-resolution depth signal. It also allows us, by coordinating with the eye tracker, to estimate the subject’s fixation point in the three-dimensional scene. To track the eyes, we use the Pupil Labs binocular eye-tracker (Kassner et al., 2014). To track the subject’s head and body motion, we use two Intel RealSense T265 tracking sensors (Grunnet-Jepsen et al., 2019) (Fig. 2). One is mounted on the subject’s back to measure body position and motion. The other is mounted on the head, attached rigidly to the headband, to measure head position and motion.

At full resolution and framerate, the total data flow produced is substantial at ${\sim }700$MB/s. The XIMEA camera contributes more than 90% of this. The mini-PC, with 3TB on-board M.2 storage, allows just over an hour of recording time at the highest framerate.

Because our device pushes the framerate limits of the sensors, one challenge was minimizing dropped frames, especially in the visual sensors. The combination of image resolution and framerate settings reported in Table 1 maximizes spatial and temporal resolution without causing a significant number of dropped frames. With this configuration, frame loss is less than two frames over 2 min of data collection with the XIMEA and RealSense RGB cameras. The depth stream typically varies in its effective framerate between 70 AND 90 Hz. We handle the frame drops that do occur with up-sampling during post-processing.

### Device ergonomics

**Fig. 3:**
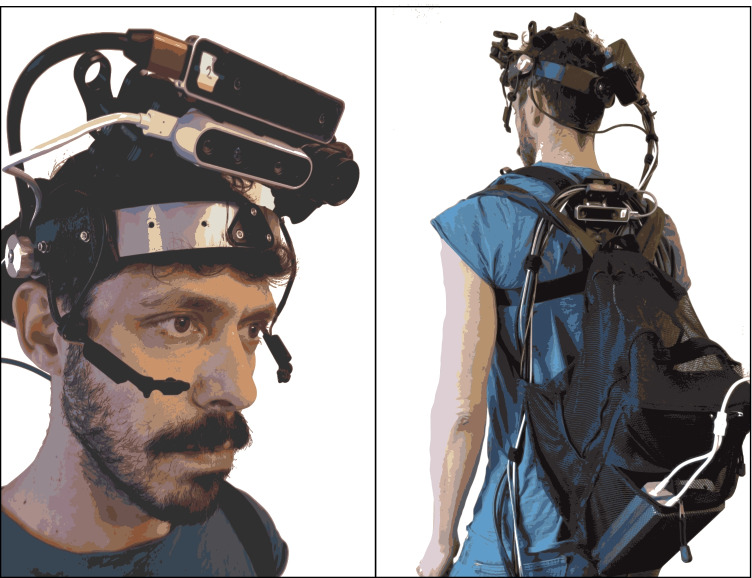
Subject wearing the device. *Left*: front view of the subject wearing the headset. The two scene cameras (XIMEA and RealSense D435i) and the tracking camera for the head (RealSense T265) are mounted together with a custom 3D-printed mount, adjustable in position with a three-point ball-and-clamp adjustable mount. Custom ball-and-socket joints combined with set screws enable positioning of eye trackers below the eyes. A white ribbon cable (not visible) connects the XIMEA camera to the rear switch box. *Right*: rear view of a subject wearing the device with the backpack in mobile configuration. Computer and batteries are housed in the backpack. XIMEA switch box is mounted with Velcro on the back of the head. Cords are bound in an adjustable loop enabling head mobility. The tracking camera (RealSense T265) for body tracking is mounted on a back strap that holds it tightly against the body with the sensor positioned just above backpack, and marked with white tape

We had two key goals in designing the head-mounted part of the device (Fig. 3): (1) to be as lightweight and comfortable as possible, and (2) to be adjustable to accommodate each participant’s head and face shape, and the task at hand. The headband is modified from a binocular indirect ophthalmoscope and adapted to hold the sensors. Custom components were designed in SolidWorks and 3D printed in PLA, making them robust yet lightweight. The three scene cameras (XIMEA, RealSense D435i, and T265) are mounted together on the same 3D-printed bracket. This is connected to the headband via three-point 3D-printed adjustable ball-and-socket joints and is secured by clamps. This arrangement enables adjustment of the pitch of the camera ensemble depending on the task. For tasks involving far viewing (e.g., outdoor walking), pitch can be adjusted upward to $\sim 0^{\circ }$, and for tasks involving near viewing (e.g., cooking) pitch can be adjusted to $\sim 30^{\circ }$ downward; mid-range tasks (e.g., seated chatting) are recorded using a mid-angle pitch. The XIMEA camera’s switch box is strapped to the back of the headband (Fig. 3). This switch box converts the PCIE connection from the computer to the ribbon-cable connection on the camera.

The two eye-tracking cameras are connected to the headband with custom designed and 3D-printed spherical joints (Fig. 3), which allow convenient, stable positioning of the camera. We anticipated degradation of eye tracking in outdoor scenes due to intense scene illumination. To deal with this, a neutral-density filter can be placed in front of the lenses when recording outdoors (Binaee, Sinnott, Capurro, MacNeilage, & Lescroart, 2021). The filter can make pupil detection more difficult, but this can be addressed when running pupil detection during post-processing.

The power and data cables connecting the sensors and computer are bound together into a clean band (Fig. 3); we loop this band behind the subject’s back with slack in the loop. The binding and slack eliminates tangling while allowing the subject to move freely. We secure the body tracker on the back using a posture-correcting strap, which is underneath the backpack, but leaves the back tracker’s cameras exposed. This avoids occluding the subject’s and camera’s views of the scene ahead, which would have occurred with front mounting.

The head mount weighs only 1.4 kg. In the future, we will investigate whether the device affects natural motion dynamics.

### Operating computer

To collect data from all sensors simultaneously, we built a PC using consumer parts (Table 2). The high-speed camera requires an x8 PCIE port for which no laptop solutions were available, so we custom-built the computer. To minimize the form factor, we use a Mini ITX motherboard with 32 GB of RAM, dual M.2 support, a PCIE port, and integrated WiFi. We use the Intel i7-8700 processor, which has sufficient computational power, yet maximizes battery life due to its low power consumption (65 W). To maintain sufficient disk-write speed and avoid RAM overflow, we use M.2 SSDs—one with 1 TB and one with 2 TB—capable of writing at 1.2 GB/s. We mounted a touchscreen inside the PC case for quick viewing and control of the computer while mobile. Power is provided by a pair of compact batteries designed to power CPAP machines. The batteries are connected in parallel and power both the computer’s DC power supply and the PCIE camera’s external power supply. We modified a standard mini ITX computer case with a custom 3D-printed enclosure. The enclosure covers the ports at the back of the computer case, exposing only the ports for DC power, an external monitor, and Ethernet, leaving the band of sensor cables permanently connected. One CPU heatsink/fan is sufficient for cooling the computer. To cool the high-speed camera, we attached two 25-mm fans to either side of the camera, powered by the camera switch box.

A video overview of the device hardware is available at: https://www.youtube.com/playlist?list=PLEloutX3oXFbi2CoA3_koqFSwKpdxLliF

**Table 2:** Details of operating computer used to control sensors and save collected data

Device	Model	Form factor	Size	Notable specs
Motherboard	Asus ROG Strix Z390-I	Mini ITX	–	Dual M.2, Wifi, PCIE
Hard drives	2x Samsung 970 Evo	M.2	1TB, 2TB	Write 1.2 GB/s
Memory	Crucial Ballistix Sport LT	DDR4 RAM	2 x 16 GB	3200 MHz
CPU	Intel i7-8700	–	–	6 Cores, 65 Watts
Batteries	BPS freedom CPAP	2x bricks (7.5”x5”x1”)	2x 100Wh	12V/8A out

## Acquisition software

### Software structure

**Fig. 4:**
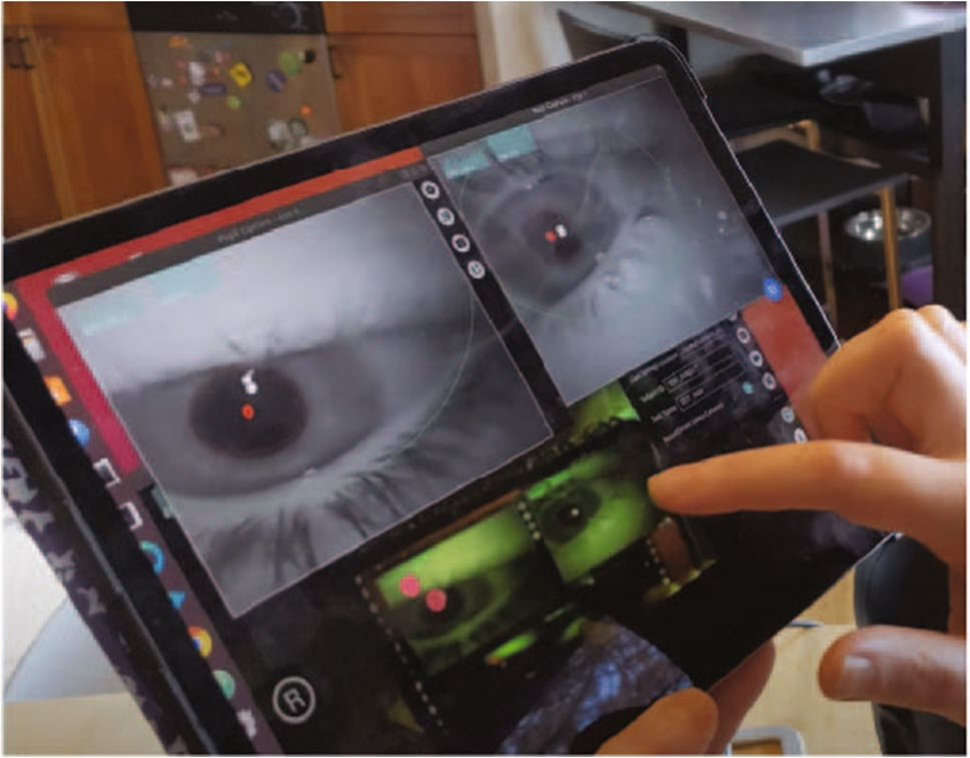
Hardware control during data collection is performed in Pupil Lab’s Pupil Capture with custom plugins running on the acquisition computer. The computer is controlled remotely with Chrome Remote Desktop over WiFi. Settings are adjusted and acquisition started and stopped by the experimenter using an iPad or laptop

We wrote the device acquisition software in Python 3 (Van Rossum & Drake, 2009) as plugins for Pupil Labs’ Pupil Capture software (Kassner et al., 2014) allowing for control of all the devices in a single graphical interface (Fig. 4). We use the RGB sensor on the Intel D435i as the world camera, and a plugin to the Pupil Capture software to save depth information as either raw *NumPy* values (Harris et al., 2020) or lossless PNG images with multiple images per file rather than the default lossy MPEG-4 encoding. Our software includes a plugin to align the RealSense depth and RGB streams online. This online alignment reduces the highest achievable framerate, so we perform spatial alignment of frames during post-processing instead. We also wrote a plugin to view and record from the XIMEA camera as well as load and apply camera settings from a YAML file. For the odometry sensors, we use the tracker code from Hausamann, Sinnott, Daumer, and MacNeilage (2021), modified slightly to support recording from both tracking devices and the Intel RGB/depth device simultaneously.

During data collection, we use the Pupil Labs’ Capture software, modified by our plugins, to observe and control the computer, switch between visual stream views, run eye-tracking calibration, adjust camera framerate and gain, and start and stop collection. When the subject’s task does not involve locomotion, we control the computer and observe the video stream using an external monitor and Bluetooth keyboard and mouse with the computer placed on a table next to the subject. During tasks involving locomotion, we control the acquisition computer through Remote Desktop over WiFi with a laptop or iPad (Fig. 4). For eye tracking, we use the default Pupil Capture eye-camera recording software, which records infrared video of each eye at 200 Hz. We turn off Pupil Capture’s online pupil detection and accomplish detection offline with the Pupil Player software after data collection is completed. This reduces the computational load on the acquisition computer, and allows manual adjustment of the pupil-detection parameters, which in turn minimizes the number of frames with failed pupil detection.

**Fig. 5:**
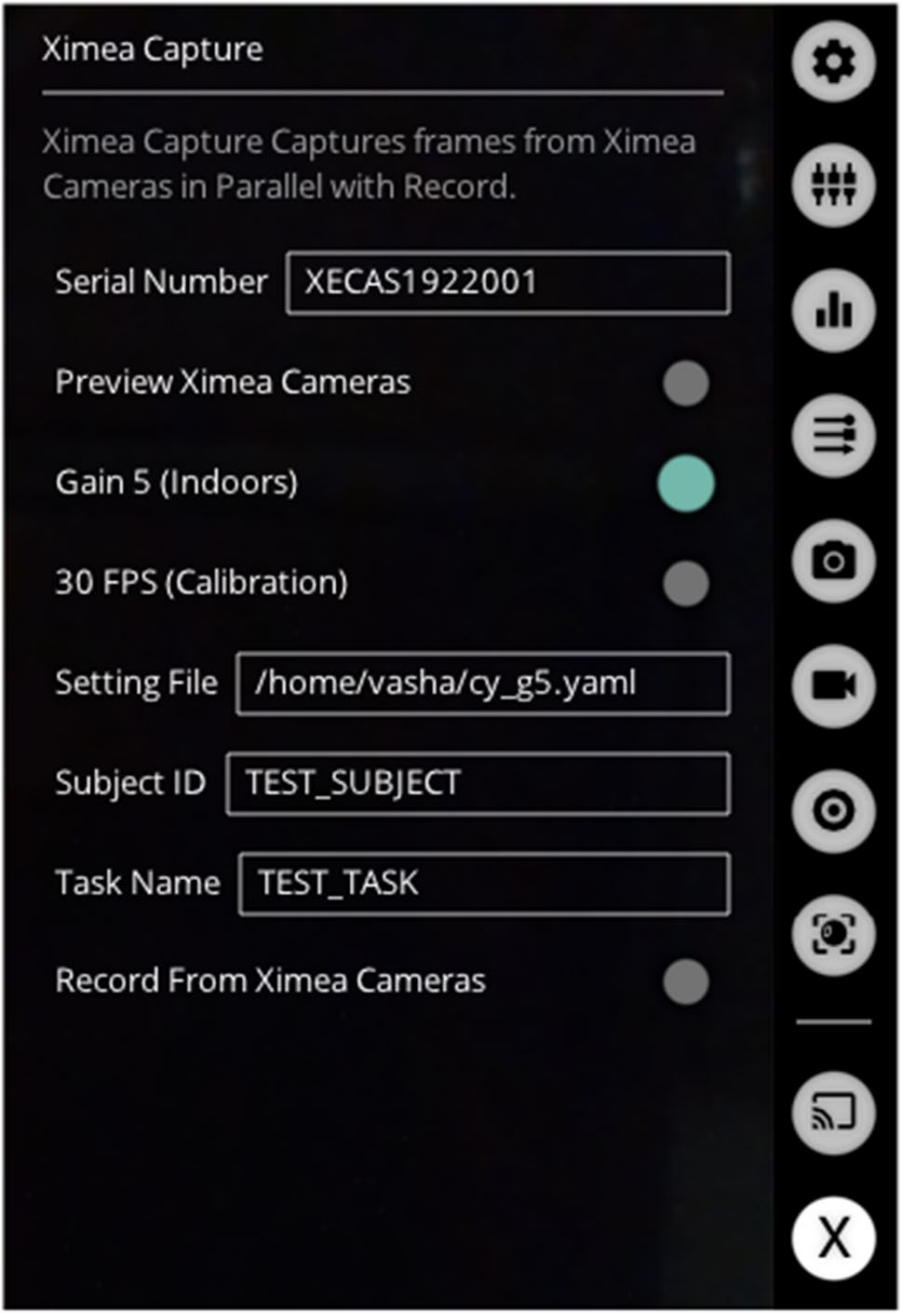
Custom plugin for recording from the high-speed XIMEA camera has a GUI interface built as a plugin for the Pupil Capture software allowing control of camera settings and recording by the experimenter

To accommodate various lighting conditions, we include an analog (sensor) gain adjustment switch for the XIMEA camera in our GUI (Fig. 5), which can be used in combination with aperture adjustment for the varying light levels in indoor and outdoor data collection. This adjustment, along with imaging a standard color checker chart (Ernst, Papst, Ruf, & Garbas, 2013), allows the experimenter to account for the system’s luminance gain and perform color balancing.

**Fig. 6:**
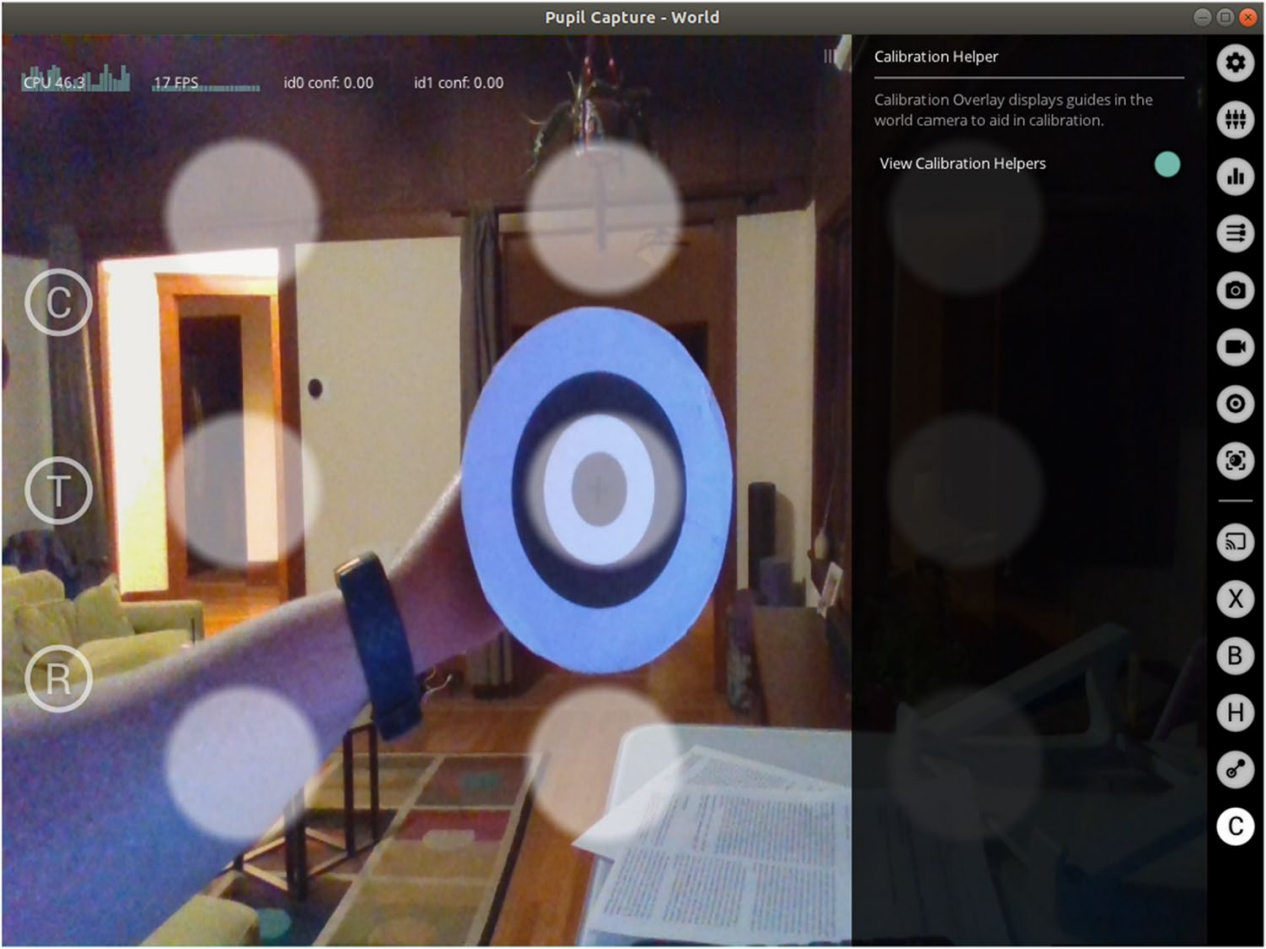
The custom Pupil Labs plugin toggles nine-point calibration positions overlaid on the video stream used for directing the subject to position a handheld calibration target for the calibration procedure

We incorporate various software scripts related to eye-tracking calibration. The high-fidelity raw image data (particularly from the XIMEA camera) is very storage intensive. To deal with this, we include a framerate adjustment switch for the XIMEA camera in our GUI. The adjustment allows us to reduce framerate during calibration, which saves storage space significantly. We also use a custom Pupil Capture plugin to visualize a nine-point marker placed within the world camera’s field of view (Fig. 6) together with a custom 3D calibration routine adapted from Gibaldi, DuTell, and Banks (2021).

### High-speed acquisition

The most significant design challenge for this system was acquiring and writing the high-speed RGB data from the XIMEA camera, particularly in accommodating the high rate of data input (637 MB/s for this sensor alone). To interface with and control the camera, configure settings, and collect data, we use xiAPI, XIMEA’s Python API. We utilize Python’s threading and queue packages to create data-collection worker threads that continuously check for and collect images and their associated timestamps from the camera’s buffer, placing them in FIFO queues. These queues are simultaneously checked by data-saving worker threads, which write queued frames and timestamps to disk. We save frames in the raw binary format from the camera (1000 images per file) for offline conversion to a standard image format. We tried other acquisition methods and they failed because either the camera’s internal buffer was overwritten due to buffer overflow or because of a buildup of frames in the computer’s RAM due to insufficient transfer of frames from RAM to disk. We use a similar multi-threaded queuing strategy for saving depth frames (also stored in raw format) to stabilize the effective framerate for the depth stream, and to avoid dropped frames.

The data collection software plugins are available at: https://github.com/vdutell/hmet_aquisition. The analysis software is available at: https://github.com/vdutell/st-bravo_analysis.

## Post-processing

After each recording session, pupil detection is performed offline inside the Pupil Player software. Then, the data are transferred to a computational server via the exposed Ethernet port for post-processing. During this offline phase, timestamp synchronization and image registration are performed to align the streams spatially and temporally. This alignment allows the depth-map and gaze-position information to be overlaid in the high-framerate camera space. Finally, we perform the remainder of the eye-tracking analysis pipeline including calibration and gaze-point estimation.

### Temporal synchronization

**Fig. 7:**
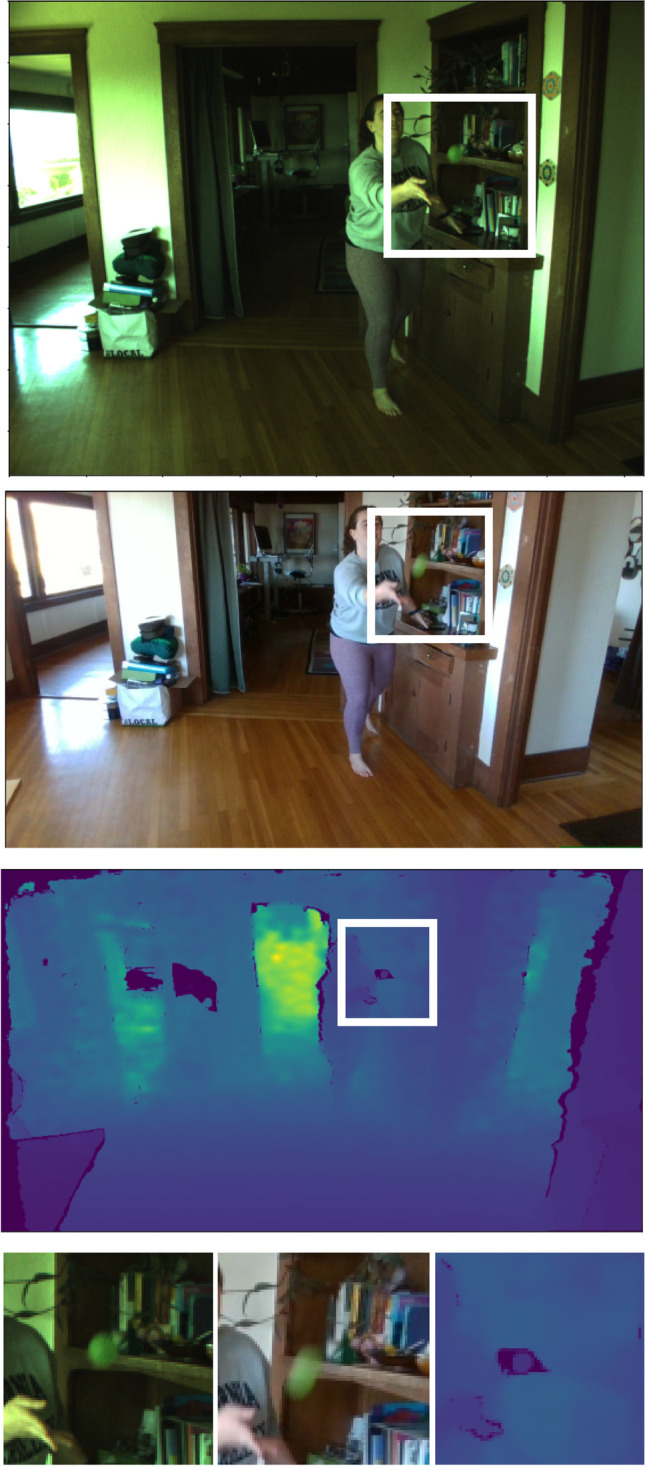
Visual streams are temporally synchronized to the framerate of the slowest visual stream (60 Hz). Temporally synchronized frames from three visual streams (*top to bottom*): XIMEA RGB stream, RealSense RGB stream, RealSense depth stream. *White boxes* indicate zoom-in on bottom panel, showing the ball in same position at moment of release from hand during toss, which is evidence that timestamps are well matched. Note the greater motion blur of the ball in the RealSense RGB stream running at 60 Hz (*bottom middle panel*) compared to the XIMEA RGB stream running at 200 Hz (*bottom left panel*)

To temporally align data from the multiple streams, we first align the timestamps of all streams (Fig. 7). Many multi-sensor devices address temporal synchronization issues with a synchronized triggering system so that timestamps are already aligned during data collection. This method is not supported in Pupil Labs, so to maximize each device’s frame-rate, we instead allow individual sensors to ‘free-run’ at their specified framerates during data collection, and then synchronize their timestamps in post-processing. For the XIMEA camera, we measure clock offsets between the sensor’s internal clock and the computer’s Unix timestamp at the beginning and end of recording. We then align the recorded timestamps to ensure there is minimal temporal drift between the two clocks during recording. For the other devices, Pupil Labs’ software handles timestamp synchronization internally with Unix timestamps directly. We investigated the accuracy of the synchronization and found that the match between cameras is within one 200-Hz frame (± 5ms) with typically fewer than one dropped frame over 2 min of data collection. In post-processing, a ground-truth timeline at the desired frame-rate is generated, and frames from each stream resampled at their nearest matching timestamp. This addresses any dropped frames and allows for resampling lower framerate streams at higher frequencies as needed.

### Spatial registration

**Fig. 8:**
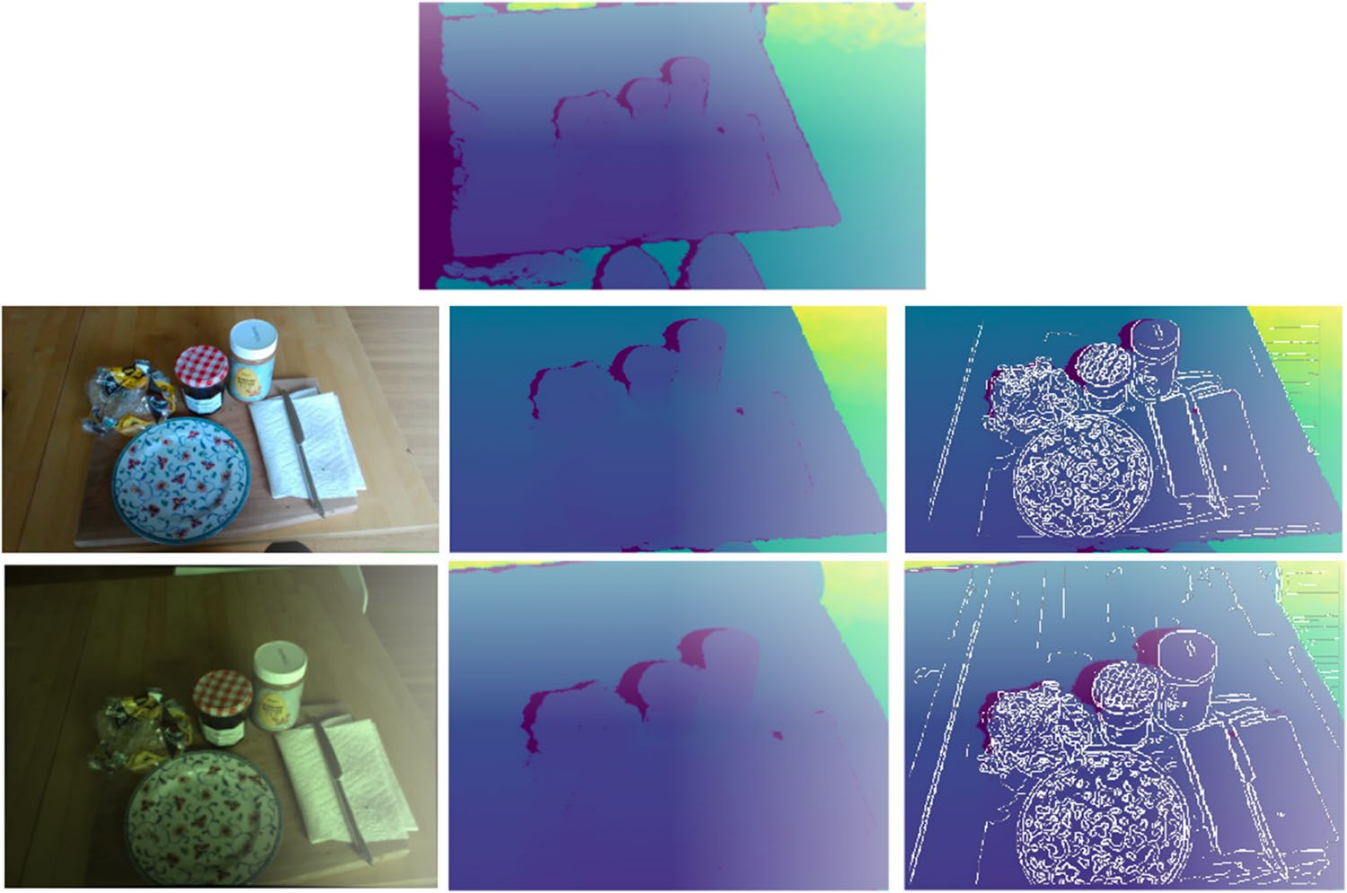
Visual streams are spatially aligned through registration with extrinsics matrices. *Top*: Original depth frame as provided by RealSense camera before spatial alignment. *Middle*: RealSense RGB frame reference (*left*) and aligned depth (*center*). *Bottom*: XIMEA RGB frame reference (*left*) and aligned depth (*center*). The right plots show the edges detected on the RGB images and superimposed on the corresponding depth maps

For spatial registration of the images, a standard offline camera and stereo calibration is combined with depth-dependent alignment. This is done twice, once for an ‘indoor’ setting with an open aperture on the XIMEA camera and once for an ‘outdoor’ setting with a smaller aperture. For each aperture setting, we first use a checkerboard grid to estimate the distortion matrix for the XIMEA camera. The RealSense RGB distortion matrix is factory calibrated and the image is undistorted on the chip. Then, we use the same checkerboard grid to run a stereo calibration, fixing the distortion matrices and estimating the extrinsics matrix between the RealSense RGB and XIMEA RGB streams. Because the rectification of the depth stream into the RGB frame of reference is depth-dependent, we use the Pyrealsense2 align_to() method to rectify the depth stream to XIMEA RGB space in two steps: 1) storing the frames in .bag file format and 2) reading in the .bag file for alignment. In the first step, we provide the RealSense camera’s self-reported depth to RGB extrinsics to the alignment method, rectifying the depth frames into the RealSense RGB camera’s frame of reference. This puts depth information into the RealSense RGB camera’s frame of reference for gaze localization. Next, we combine the RealSense to XIMEA RGB extrinsics matrix (measured during the stereo calibration) with the RealSense camera’s self-reported depth to RGB extrinsics matrix to create a depth-to-XIMEA extrinsics matrix. Finally, we used this combined RealSense RGB to XIMEA RGB extrinsics matrix in the .bag file alignment, rectifying these depth frames directly into the XIMEA camera’s frame of reference. Performing the alignment in one step with a combined extrinsics matrix avoids loss of image data due to the vertical field of view of the RealSense RGB being smaller than the depth and XIMEA RGB streams. We perform all spatial registration offline after data collection. Figure 8 shows an example set of aligned frames. The plots on the right report the edges detected on the RGB images superimposed on the corresponding depth maps, and serve to verify the correctness of the alignment.

## Accuracy

### Spatial

There are various sources of error within the sensors, their synchronization, and eye-tracking calibration that individually contribute to the overall spatial and temporal accuracy limits of the system. The largest source of spatial uncertainty in our system is in the eye tracking. We use a custom, depth-aware calibration and gaze-localization method, which reduces estimated error to 0.25^∘^ in the best case, and 0.5–0.6^∘^ for an average subject. This is smaller than the < 1^∘^ and the 1.5–2.5^∘^ accuracy reported for the Pupil Labs 2D and 3D gaze mapping methods, respectively (Kassner et al., 2014). We report the details of this custom method in previous work (Gibaldi, DuTell, & Banks, 2021). With the magnification factor of the lens used in our system, 0.25^∘^ corresponds to approximately eight pixels; a wider angle lens would reduce the pixel error, and a temporal smoothing window could also be applied to the eye trace to reduce high-frequency jitter.

### Temporal

The largest source of temporal uncertainty in our system is in the depth stream, which is framerate limited by maximum sampling rate of the RealSense depth sensor of 90 Hz. We up-sample the depth stream in post-processing from the native 90-Hz to the 200-Hz sampling rate of the high-fidelity cameras. Because our gaze mapping and spatial re-projection methods are depth-dependent, this depth accuracy limitation propagates through our analysis and is a limiting factor of the system.

### Odometry

In addition to accuracy limitations in the visual sensors, the system’s estimate of the head and body positions and velocities are limited by the accuracy and precision of the RealSense T265 sensors. The accuracy of this sensor has been thoroughly evaluated in the literature for robotics (Alapetite, Wang, Hansen, Zajaczkowski, & Patalan, 2020), and in a head-mounted context similar to ours (Hausamann, Sinnott, Daumer, & MacNeilage, 2021), where errors tend to accrue over time. Accuracy is best for slow walking in small- to medium-sized environments (< 5% error in speed, < 10% error in trajectory length when walking in a hallway) but suffers when the subject is running, and navigating larger environments ($\sim$25% error in speed, $\sim$20% error in trajectory length for running in an outdoor courtyard). Given our device utilizes two T265 sensors, some post-processing could use this partial redundancy to address re-position errors and temporal drift, but we have not yet implemented this. It is important to note that these accuracy limitations in the T265 sensors affect only the head and body odometry signal, and therefore for most use cases, have no effect on the spatiotemporal resolution of the visual streams. This is because gaze localization is performed independently with only the D435i, XIMEA, and Pupil Labs sensors. Such error accrual in the T265 sensor would, however, need to be considered in the case that gaze localization is desired in a world (non-head) reference frame, where head and/or body pose tracking is used.

## Discussion

To our knowledge, the apparatus and data collection and analysis methods are novel in their enabling of uncompressed, high-fidelity, data-intensive, and synchronized multi-sensor signal capture in a mobile eye-tracking device. Our device enables a high-quality reconstruction of the natural visual input as experienced by the human eye as a subject goes about everyday activities. At the same time, it records the subject’s body, head, and eye movements.

While our device has been optimized for minimal weight on the head and body, the unavoidable weight and bulk of the device is a limitation. The high-frame-rate camera (particularly in its switch-box/PCIE system) adds weight and bulk to the head, which may restrict subject movement during data collection, and may affect the statistics of the measured body, head, and eye motion as has been previously reported for head-mounted displays (Knight & Baber, 2007). When we designed the device, this camera was the best available option for high-speed collection without introducing artifacts due to on-camera compression. Since then, new cameras have been released that connect directly to a computer via other methods including USB-C. As such they enable high-fidelity and lighter weight.

The weight in the backpack can also affect the biomechanics of standing and walking (Devroey, Jonkers, De Becker, Lenaerts, & Spaepen, 2007). Given that head, body, and eye motion are linked (Imai et al., 2001), the weight of our acquisition computer may affect the statistics of a subject’s head, eye, and body motion. Consequently, in the future we intend to determine how the device affects measured statistics.

There are a number of contexts in which our device is ideally suited for data collection, due to design considerations that prioritize uncompressed, high spatial and temporal resolution visual data, yet allow a reasonable degree of mobility. This high-fidelity visual stream data coupled with gaze localization and corresponding depth data allows for reconstruction of the signal seen by the retina in natural conditions. Combined with odometry data, this system can be used to study the role of environmental, body, head, and eye movement (and their coordination) in generating this dynamic retinal image. Potential avenues of exploration include the predictability of head and eye motion from environmental features and motion, differences in motion statistics between foveal and peripheral vision, and the role of binocular vision and gaze stabilization in locomotion.

Studying specific features of human oculomotor behavior in the natural environment necessitates this type of high-fidelity system. This is especially true for studying smooth pursuit, saccades, and vergence. High temporal fidelity is also crucial for studies of the vestibulo-ocular reflex (VOR), and finally for high-accuracy fixation localization. Our system enables these studies to occur outside the laboratory under more natural conditions. In studying hand/eye motor coordination, high temporal fidelity is also critical in situations where fast motion is present such as sports, although the weight and bulk of the system limits the use of this device in the most active sports. Finally, in addition to the specific aspects of human visual behavior that can be well studied with our system, many methods of downstream data analysis methods such as Fourier analysis, optic flow, and spatiotemporal motion saliency, require and/or are aided by the high-fidelity, compression-free data collection offered by our system.

Data collected with this device reveal the complex spatiotemporal patterns of light that strike the retina during everyday life. Quantifying the statistics of these patterns will be important for gaining a better understanding of the human visual and motor systems and how they have adapted to the natural environment. The data collected with this device will be useful to a number of scientific and technical communities including vision science, experimental psychology, neuroscience, bioengineering, computer science, and display technology.
